# Supplementary material for: Inhibitory Effects of Antipsychotic Chlorpromazine on the Survival, Reproduction and Population Growth Other Than Neurotransmitters of Zooplankton in Light of Global Warming
Source: Int J Environ Res Public Health. 2022 Dec 2;19(23):16167. doi: 10.3390/ijerph192316167 (PMC9736287; doi:10.3390/ijerph192316167)
Supplement: Supplementary file 1 [file ijerph-19-16167-s001.zip › ijerph-1996066 supplementary_1.pdf]

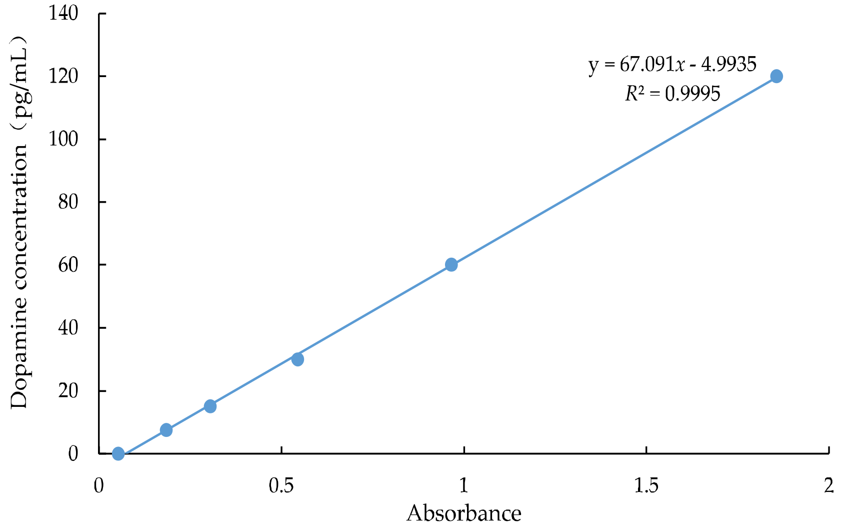

Figure S1 Standard curve for dopamine ELISA kit.

Table S1 Results of the variance (two-way ANOVA) performed on the selected life history variables and dopamine concentration of *B. calyciflorus* in relation to chlorpromazine concentrations in three temperatures. *SS* = sum of squares, *df* = degrees of freedom, *MS* = mean squares, and *F* = *F*-ratio.

**Sources of Variation**	***SS***	***df***	***MS***	***F***	***p***
Life expectancy at hatching					
Temperature (A)	5452.080	2	2726.040	146.561	3.530×10^{-14} ***
CPZ concen. (B)	2023.800	3	674.600	36.269	4.462×10^{-9} ***
A × B	13.200	6	2.200	0.118	0.993
Error	446.400	24	18.600		
Net reproductive rate					
Temperature (A)	255.277	2	127.639	97.537	2.988×10^{-12} ***
CPZ concen. (B)	227.943	3	75.981	58.062	3.777×10^{-11} ***
A × B	16.623	6	2.770	2.117	0.089
Error	31.407	24	1.309		
Generation time					
Temperature (A)	2627.641	2	1313.820	296.722	1.190×10^{-17} ***
CPZ concen. (B)	221.034	3	73.678	16.640	5.000×10^{-5} ***
A × B	31.642	6	5.274	1.191	0.344
Error	106.267	24	4.428		
Intrinsic rate of population increase					
Temperature (A)	0.008	2	0.004	952.031	2.560×10^{-4} ***
CPZ concen. (B)	0.001	3	2.560×10^{-4}	59.031	3.175×10^{-11} ***
A × B	8.137×10^{-5}	6	1.356×10^{-5}	3.126	0.021 *
Error	1.040×10^{-4}	24	4.338×10^{-6}		
Dopamine concentration					
Temperature (A)	8.070	2	4.035	6.490	0.006 **
CPZ concen. (B)	125.792	3	41.931	67.446	7.733×10^{-12} ***
A × B	1.825	6	0.304	0.489	0.810
Error	14.921	24	0.622		

* $p < 0.05$, ** $p < 0.01$, and *** $p < 0.001$
